# A comprehensive high-throughput detection strategy: an indirect ELISA for serodetection of the novel aMPV/C in ducks

**DOI:** 10.3389/fmicb.2026.1973341

**Published:** 2026-09-25

**Authors:** Lin Lin, Shen Cao, Nansong Jiang, Longfei Cheng, Hongmei Chen, Yongfu Liao, Jinhua Liu, Rongchang Liu, Chunhe Wan, Yu Huang, Qizhang Liang, Weiwei Wang, Guanghua Fu, Qiuling Fu

**Affiliations:** 1 Institute of Animal Husbandry and Veterinary Medicine, Fujian Key Laboratory for Control and Prevention of Avian Diseases, Fujian Industry Technology Innovation Research Academy of Livestock and Poultry Diseases Prevention and Control, Fujian Academy of Agricultural Sciences, Fuzhou, China; 2 Changting County Bureau of Agriculture and Rural Affairs, Longyan, China; 3 College of Veterinary Medicine of China Agricultural University, Beijing, China

**Keywords:** avian metapneumovirus subtype C, novel, ducks, indirect ELISA, serological detection

## Abstract

**Introduction:**

Avian metapneumovirus subtype C (aMPV/C), a re-emerging and widespread epidemic in China, causes hydrosalpinx fluid syndrome (HFS) in laying ducks, resulting in significant economic losses to the poultry industry. In the absence of any licensed vaccines or antiviral drugs for aMPV/C, accurate diagnosis and timely eradication of the infected ducks remain the primary control measures, underscoring the urgent need for reliable and effective detection methods.

**Methods:**

In this study, the purified whole novel aMPV/C was used to develop an indirect enzyme-linked immunosorbent assay (iELISA) for the detection of aMPV/C antibodies in ducks.

**Results:**

The cut-off value of the iELISA was determined to be 0.258. No cross-reactivity was observed with duck sera positive for antibodies against H5 AIV, NDV, DTMUV, BTV, DHAV-3, DPV, EDSV, MDPV, or GPV, demonstrating good specificity. Both intra-assay and inter-assay coefficients of variation were less than 10%, and the detection sensitivity of the iELISA reached a serum dilution of 1:102400. A total of 216 clinical serum samples from three duck breeds were tested *via* the established iELISA, of which 129 (59.7%) were positive. The concordance rate between the iELISA and an indirect immunofluorescence assay (IFA) was 100% (53/53).

**Conclusion:**

Overall, this cost-effective iELISA provides a reliable tool for the detection of aMPV/C antibodies in clinical diagnostics and epidemiological surveillance.

## Introduction

Avian metapneumovirus (aMPV) was first identified in South Africa in 1978 (Buys and Preez, 1980) and has since become a globally distributed pathogen, infecting both domestic poultry and wild avian species (Salles et al., 2023). The virus causes upper respiratory disease, typically characterized by sinusitis, head swelling, and decreased egg production (Clontz and Hoerr, 2026; De Graaf et al., 2009). In uncomplicated cases, mortality is generally low (2–5%); however, co-infections with secondary pathogens can elevate mortality to as high as 25%. Based on genetic and antigenic variability, aMPV is classified into four subtypes (A-D) (Juhasz and Easton, 1994; Bäyon-Auboyer et al., 2000; Rima et al., 2017). Subtypes A and B are distributed worldwide, with subtype B predominating in Europe and China (Franzo et al., 2020). Subtype C naturally infects turkeys, chickens, ducks, and wild birds, and its genome exhibits the highest homology with human metapneumovirus (Hatfield et al., 2026). Prior to 2025, aMPV/C was known to comprise two distinct lineages: the North American and the Eurasian lineages (Quan et al., 2020). Recently, our group identified a novel lineage circulating in Sheldrake ducks (Fu et al., 2025). In contrast, subtype D has only been reported in France (Umar et al., 2019). Notably, two novel and divergent aMPV strains were detected in North America in 2019, further highlighting the ongoing expansion of aMPV genetic diversity (Canuti et al., 2019; Retallack et al., 2019).

In China, aMPV/C was first recorded in Muscovy ducks in 2014, with no subsequent isolations from waterfowl reported until 2021 (Sun et al., 2014). Since then, the virus has become increasingly prevalent nationwide, causing hydrosalpinx fluid syndrome (HFS) in laying ducks and respiratory disease in meat ducks (Yu et al., 2024; Xu et al., 2025; Fu et al., 2025). In 2025, we isolated a novel strain, designated aMPV-FJ21, from Sheldrake ducks exhibiting HFS (Fu et al., 2025). Molecular characterization revealed that this strain is genetically distinct from both the North American and Eurasian lineages, indicating ongoing genetic diversification and adaptive evolution of aMPV/C in China (Fu et al., 2025). These findings underscore the urgent need for effective control measures, particularly rapid and reliable diagnostic tools.

Current diagnostic approaches for aMPV/C include pathogen isolation, molecular assays, and serological tests. Virus isolation is labor-intensive and time-consuming, and its recovery rate from clinical samples is notoriously low, reported as only 8% (2/25), rendering it impractical for routine clinical diagnosis (Kwon et al., 2010; Sun et al., 2014). Molecular methods, such as PCR, offer high specificity and sensitivity but require specialized equipment and skilled personnel (Shin et al., 2000). Moreover, their diagnostic utility is constrained by the short viremic window of aMPV/C, typically lasting only 2–10 days post-infection (Cook et al., 1993; Cook et al., 2001). Serological assays, including virus neutralization tests (VNT), IFA, and ELISA, are widely employed for antibody detection (Chiang et al., 2000). Among these, ELISA is particularly suitable for large-scale surveillance due to its high throughput, sensitivity, and specificity, and remains a cornerstone for monitoring infection pressure, assessing flock immunity, and guiding vaccination strategies (Saeed et al., 2026). However, to date, no commercial ELISA kit is available for the detection of aMPV/C antibodies in duck sera.

To bridge this diagnostic gap, we developed an iELISA using a purified novel aMPV/C isolate as the coating antigen, specifically designed for the detection of aMPV/C antibodies in duck sera. This assay offers a practical and reliable tool for the diagnosis and epidemiological surveillance of aMPV/C infection in ducks, with the potential to support disease control efforts in affected regions.

## Materials and methods

### Virus purification

The aMPV/C isolate (aMPV-FJ21, 10^6.56^ TCID_50_/mL) maintained in our laboratory was used in this study (Fu et al., 2025). Vero cells were inoculated with the virus, maintained in serum-free medium, and harvested at 96 h post-infection (hpi), followed by storage at −70 °C. The harvested cells were subjected to three cycles of freezing and thawing. The virus was then inactivated with 0.1% formaldehyde at 37 °C overnight. For antigen purification, 300 mL of inactivated suspension was clarified by centrifugation at 10000 × g for 15 min at 4 °C to remove cellular debris. Subsequently, the virus suspension was pelleted by ultracentrifugation at 45000 × g for 5 h. The resulting pellet was resuspended in 1 mL of 0.01 M phosphate-buffered saline (PBS, pH7.4) and used as the coating antigen for ELISA development. Meanwhile, the purified virus was examined using a transmission electron microscope (TEM, H-7500, Hitachi, Japan). And its protein concentration was determined using the enhanced BCA protein assay kit (Beyotime Biotechnology China, Fuzhou, China).

### Generation of polyclonal antibody against aMPV/C

Purified aMPV-FJ21 strain, emulsified with an equal volume of Freund’s complete adjuvant (Sigma-Aldrich, Kenilworth, United States), was used as the immunogen for polyclonal antibody production. Sheldrake ducks were immunized with 1 mL immunogen *via* multiple subcutaneous injections in the dorsal region, with a negative control group included in parallel. Immunizations were administered at 2-week intervals for a total of four doses. The primary immunization used Freund’s complete adjuvant, whereas the three subsequent booster doses employed Freund’s incomplete adjuvant. Serum samples were collected 2 weeks after the final immunization for subsequent analysis.

### Establishment of the whole aMPV/C virus-based iELISA

#### Checkerboard titration

Optimal iELISA conditions were determined by checkerboard titration. Briefly, 96-well plates (Corning, New York, United States) were coated with 100 μL/well of purified aMPV-FJ21 at concentrations of 16, 8, 4, 2, 1 and 0.5 μg/mL in carbonate buffer (CBS, 0.05 M, pH 9.6) at 37 °C for 1 h. After three washes with PBS containing 0.05% (v/v) Tween 20 (PBST), plates were blocked with 10% skimmed milk in PBS at 37 °C for 2 h, followed by three additional washes. aMPV/C-positive and aMPV/C-negative duck sera, serially diluted from 1:100 to 1:3200 in PBS, were added (100 μL/well) and incubated at 37 °C for 1 h. Plates were washed, and 100 μL of horseradish peroxidase-conjugated goat anti-duck IgG antibody (1:10000, KPL, Maryland, United States) was added, followed by incubation at 37 °C for 1 h. After washing, 100 μL of TMB substrate (Solarbio, Beijing, China) was added and incubated at 37 °C for 10 min. The reaction was stopped with 50 μL/well of stop solution, and the optical density was measured at 450 nm (OD_450nm_) using an automatic ELISA reader (SpectraMax M3, Molecular Devices, USA). The optimal conditions were selected based on the highest positive-to-negative (P/N) ratio.

### Optimization of experimental parameters

To determine optimal coating conditions, different coating times (overnight at 4 °C; 1, 2 and 3 h at 37 °C) and coating solutions (normal saline, PBS, CBS, and ddH_2_O) were evaluated. The iELISA was then performed to optimize blocking times (60, 90, 120, and 150 min at 37 °C) and blocking solutions (1, 2% BSA, 5, 10% skimmed milk, and 10% FBS). Next, antibody–antigen reaction times, various incubation periods (30, 60, 90, and 120 min at 37 °C) and dilutions of the enzyme-labeled secondary antibody (1:2000, 1:4000, 1:5000, 1:8000, and 1:10000) were tested. Finally, TMB reaction times (5, 10, 15, 20, and 25 min at 37 °C) was evaluated to identify the optimal assay conditions.

### Determination of the cut-off value

To determine the cut-off value for the iELISA, 57 serum samples from healthy ducks (negative for aMPV/C) were used. The cut-off was calculated based on the mean and standard deviation (SD) of the OD_450nm_ values obtained from negative samples. It was defined as the mean OD_450nm_ of the 57 negative sera plus 3SD, a threshold that theoretically provides >99% confidence that results from true negative samples fell below this value.

### Specificity and repeatability

The specificity of the iELISA was evaluated using positive sera against H5 avian influenza virus (H5 AIV, titer: 7log2), Newcastle disease virus (NDV, titer: 6log2), egg drop syndrome virus (EDSV, titer: 6log2), duck Tembusu virus (DTMUV, titer: 1:512), duck plague virus (DPV, **t**iter: 1:640), Batai virus (BTV, titer: 1:256), Muscovy duck parvovirus (MDPV, titer: 1:512), goose parvovirus (GPV, titer: 1:640), and duck hepatitis A virus type 3 (DHAV-3, titer: 1:1024). Antibody titers against H5 AIV, NDV, and EDSV were assessed using hemagglutination inhibition (HI) assay, whereas titers against DTMUV, DPV, BTV, MDPV, GPV, and DHAV-3 were evaluated by iELISA. aMPV/C-positive and aMPV/C-negative sera were included as controls.

To assess the repeatability of the iELISA, four aMPV/C-positive and four aMPV/C-negative sera were randomly selected and tested in triplicate on both the same batch and different batches of ELISA plates. Variability was expressed as the coefficient of variation (CV), calculated as the standard deviation (SD) divided by the mean optical density at OD_450nm_ for each sample group.

### Sensitivity of the iELISA

The sensitivity of the iELISA was evaluated using twofold serial dilutions of three aMPV/C-positive serum with different OD_450nm_, ranging from 1:100 to 1:204800, in a parallel assay.

### IFA

The viral antigen was detected by IFA during this period as described by Fu et al. (2025). Briefly, Vero cells in 96-well plates (10^5^ cells/well) were infected with the aMPV-FJ21 strain (10^5^TCID_50_) for 72 h. Following infection, cells were fixed with pre-cooled 4% paraformaldehyde (Sigma-Aldrich, Kenilworth, USA) in PBS for 15 min at 4 °C and permeabilized with 0.1% Triton X-100 (Sigma-Aldrich, Kenilworth, USA) in PBS containing 2% BSA (Beyotime, Shanghai, China) for 30 min. The fixed cells were then incubated with test serum (1:100) as the primary antibody for 1.5 h at 37 °C. aMPV/C-negative serum served as a mock control. After washing with PBST, the cells were incubated with Alexa Fluor 488-labeled goat anti-duck IgG (1:200, KPL, Maryland, United States) for 1 h at 37 °C. Finally, all samples were observed under a Nikon A1R confocal fluorescence microscope (Nikon Instruments, Inc., Melville, United States).

### Detection of antibodies in clinical serum samples

A total of 216 clinical serum samples from ducks exhibiting a drop in egg production or respiratory symptoms, comprising 136 from Sheldrake ducks, 42 from Muscovy ducks, and 38 from Cherry Valley ducks, were tested using the established iELISA. Subsequently, 53 of these samples were randomly selected for detection by IFA, and the concordance rate between the two methods was calculated.

### Statistical analysis

Each experiment was carried out in at least three independent replicates. Significant differences were determined using *t*-tests and non-parametric tests in GraphPad 8.0, with a significance level set of *p* < 0.05.

## Results

### Virus purification and identification

The harvested virions were further purified by ultracentrifugation and TEM revealed that the virions exhibited irregular, spherical, and long filamentous morphologies, with some filamentous particles reaching approximately 1,000 nm in length (Figure 1). This morphology was consistent with the characteristic appearance of aMPV/C, confirming the successful purification of intact virions. The concentration of the purified virus was 2.05 mg/mL determined by BCA.

**Figure 1:**
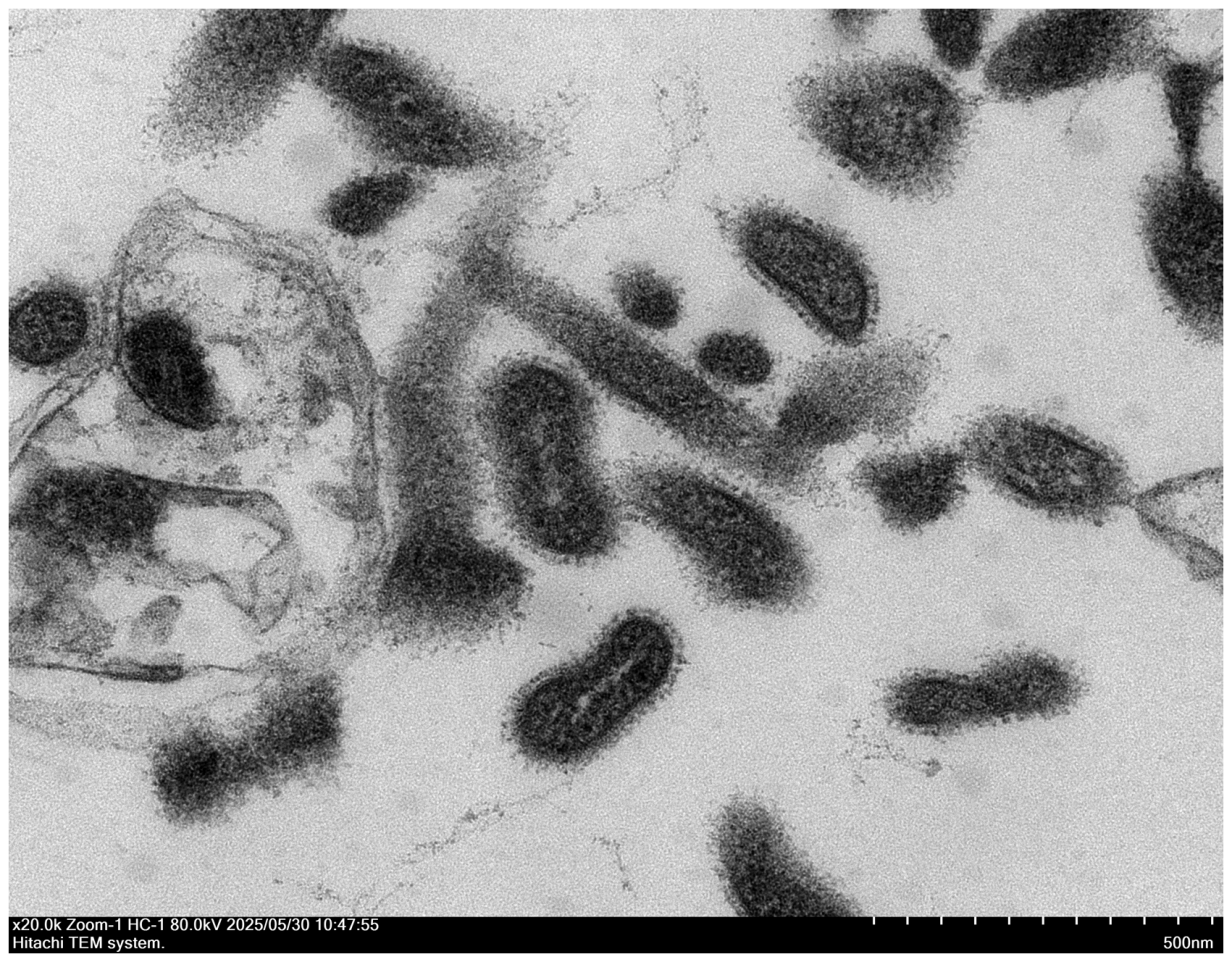
The electron micrograph of the purified aMPV-FJ21. Spherical, irregular, long filamentous and enveloped particles (20 nm ~ 500 nm in diameter) were observed.

### Development of iELISA for detection of aMPV/C antibodies

An iELISA was developed for aMPV/C antibody detection, and all reaction conditions were systematically optimized. The optimal coating antigen concentration as determined by checkerboard titration was 4 μg/mL, and the optimal serum dilution was identified as 1:1600 (Figure 2A). For coating and blocking, the optimal protocol consisted of coating with CBS for 1 h at 37 °C (Figures 2B,C) and blocking with 10% skimmed milk for 90 min at 37 °C (Figures 2D,E). Further optimization established the following conditions: 60-min serum incubation (Figure 2F), a secondary antibody dilution of 1:4000 with a 60-min incubation (Figures 2G,H), and a 20-min TMB (Figure 2I).

**Figure 2:**
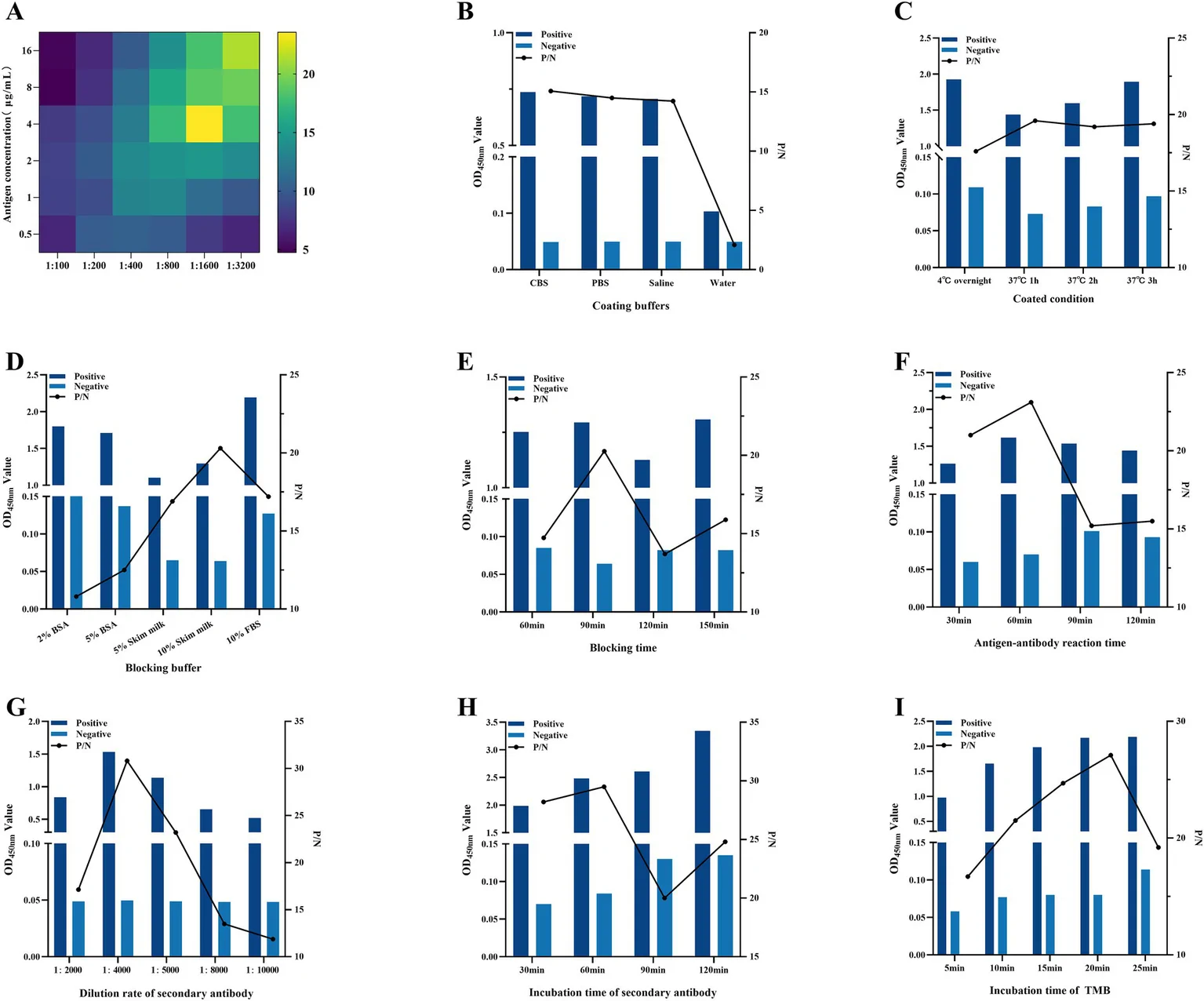
Optimization of working conditions of the established iELISA. **(A)** The optimal concentration of the coating antigen and the dilution duck serumdetermined *via* the checkerboard titration. **(B,C)** Determination of the optimal coating buffer and condition. **(D,E)** Determination of optimal blocking buffer and time. **(F)** Determination of the antigen–antibody incubation time. **(G,H)** Optimization of secondary antibody dilution and reaction time. **(I)** Optimal incubation time of TMB.

### Cut-off values for the iELISA

To establish the cut-off value for the iELISA, 57 healthy duck serum samples were tested. The mean OD_450nm_ value of the negative samples was 0.123, with a SD of 0.045. Based on these data, the cut-off value was set at 0.258 (mean + 3SD). Accordingly, serum samples with an OD_450nm_ value≥0.258 were considered positive for aMPV/C antibodies, whereas those with values below this threshold were classified as negative (Figure 3).

**Figure 3:**
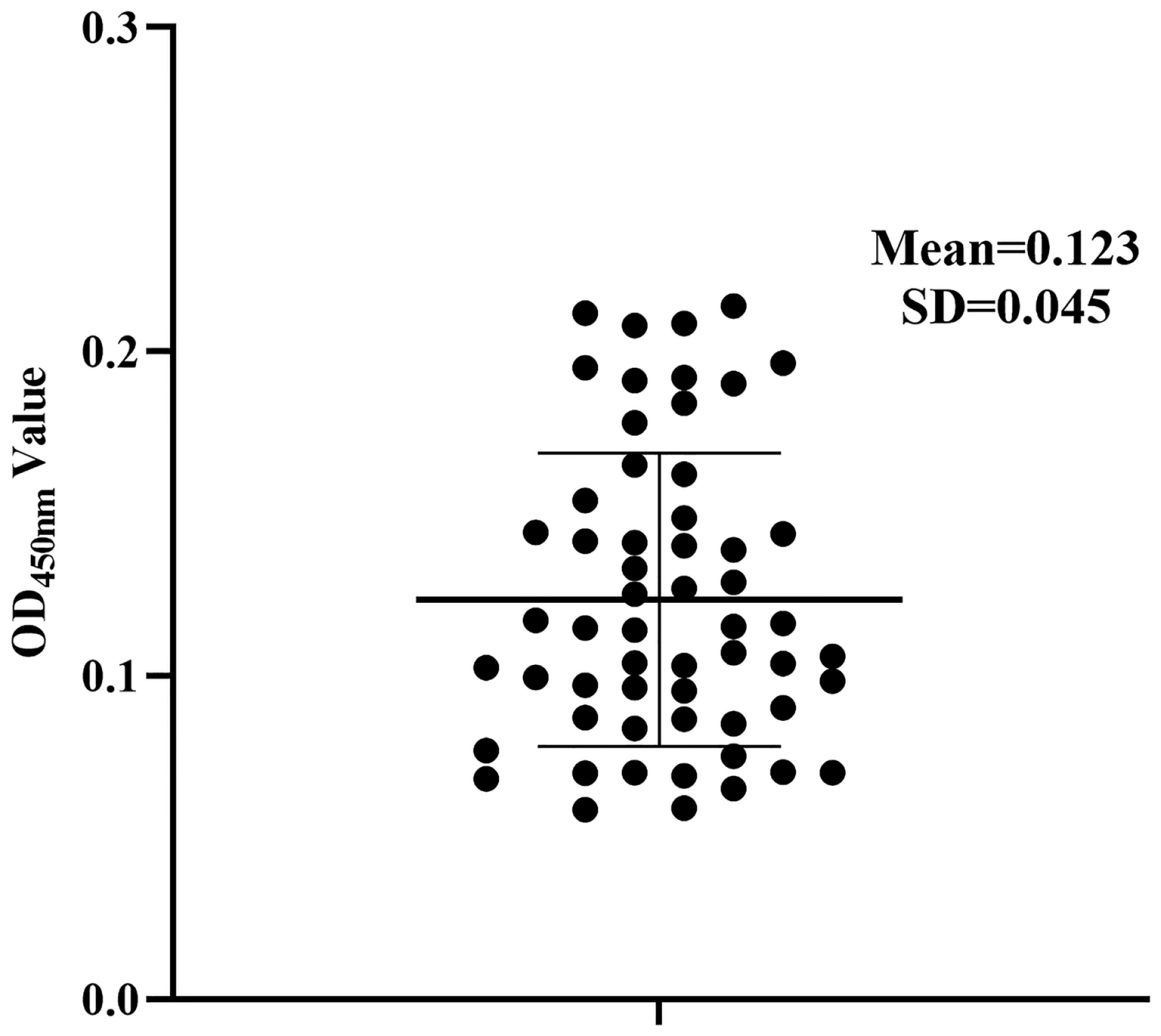
Determination of the cut-off value in iELISA method. A total of 57 negative sera were detected *via* the iELISA and the cut-off value was set at 0.258 (mean + 3SD).

### Specificity, reproducibility, and sensitivity of iELISA

Specificity testing showed that only aMPV/C-positive sera reacted in the iELISA, whereas sera against H5 AIV, NDV, EDSV, DTMUV, DPV, BTV, MDPV, GPV, and DHAV-3 were all negative (Figure 4A). Repeatability was assessed using eight selected serum samples. Inter-assay CVs ranged from 1.42 to 4.48%, and intra-assay CVs ranged from 1.28 to 6.74%, both below the acceptable threshold of 10% (Table 1), indicating excellent reproducibility. Sensitivity was determined by testing three aMPV/C-positive sera in twofold serial dilutions from 1:100 to 1:204800. The highest dilution yielding a positive signal was 1:102400 (Figure 4B). These results demonstrate that the iELISA exhibits high specificity, reproducibility, and sensitivity for detecting aMPV/C antibodies.

**Figure 4:**
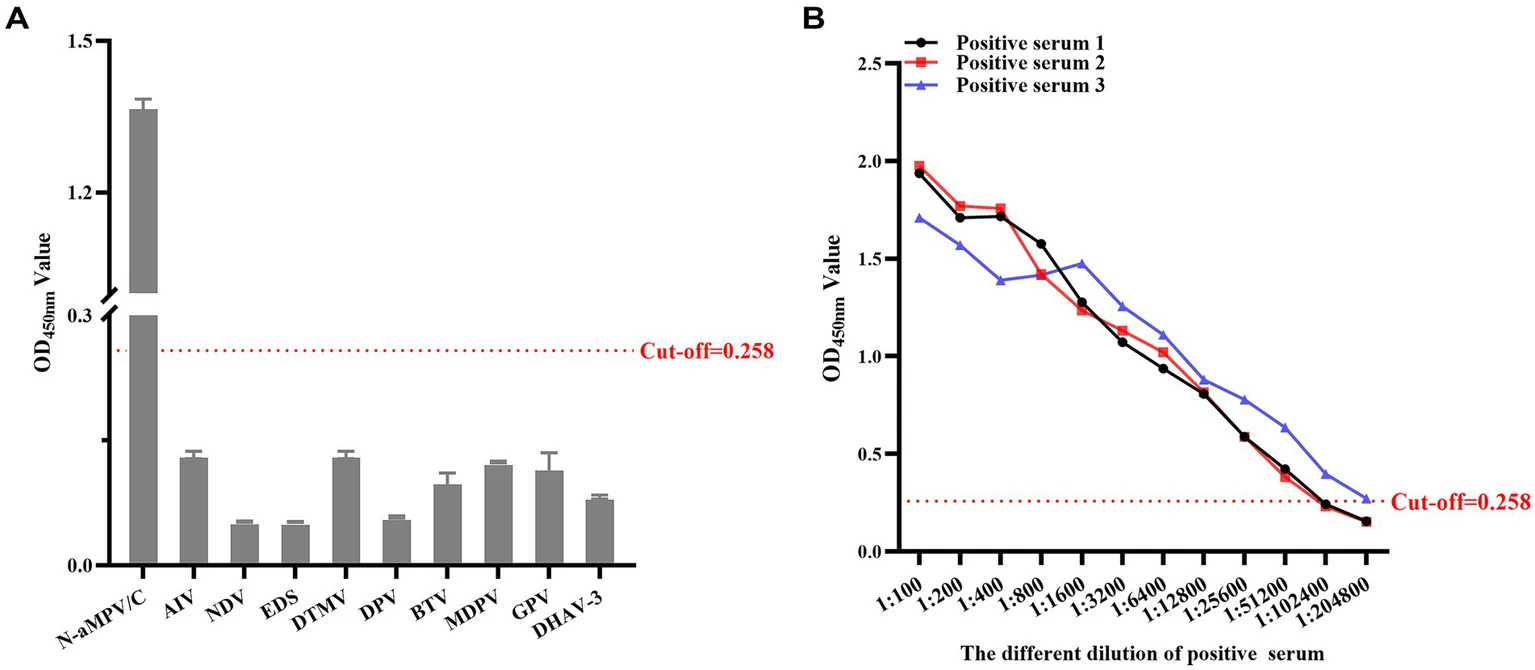
Cross reaction and detection limit of the iELISA. **(A)** The cross-reactions of the iELISA were assessed by detecting 10 sera against common duck viruses. **(B)** The lowest detection limit of the iELISA was determined by serial dilutions of three duck aMPV/C-positive serum samples.

**Table 1:** Results of the repeatability test of the iELISA.

Serum samples	Intra-assay		Inter-assay	
	Mean ± SD	CV (%)	Mean ± SD	CV (%)
Positive serum 1	2.127 ± 0.095	4.48%	2.145 ± 0.062	2.90%
Positive serum 2	2.447 ± 0.074	3.01%	2.486 ± 0.049	1.95%
Positive serum 3	2.245 ± 0.032	1.42%	2.271 ± 0.029	1.28%
Positive serum 4	2.315 ± 0.062	2.69%	2.364 ± 0.042	1.77%
Negative serum 1	0.079 ± 0.002	2.23%	0.08 ± 0.003	3.26%
Negative serum 2	0.108 ± 0.003	3.21%	0.112 ± 0.004	3.63%
Negative serum 3	0.204 ± 0.005	2.43%	0.201 ± 0.001	5.20%
Negative serum 4	0.104 ± 0.002	1.78%	0.101 ± 0.007	6.74%

### Detection of antibodies in clinical serum samples

A total of 216 duck serum samples were analyzed using the established iELISA, with results validated by IFA (Figure 5). As presented in Table 2, the positivity rate was highest in Sheldrake ducks (69.1%, 94/136), followed by Muscovy ducks (57.1%, 24/42) and Cherry Valley ducks (28.9%, 11/38), resulting in an overall seroprevalence of 59.7% (129/216). Furthermore, among 53 randomly selected samples, 45 were concordantly identified as aMPV/C-positive and 8 were negative by iELISA and IFA (Table 3). The concordance rate between the two detection methods was 100% (53/53), indicating that the iELISA is a reliable and rapid tool for clinical diagnosis.

**Figure 5:**
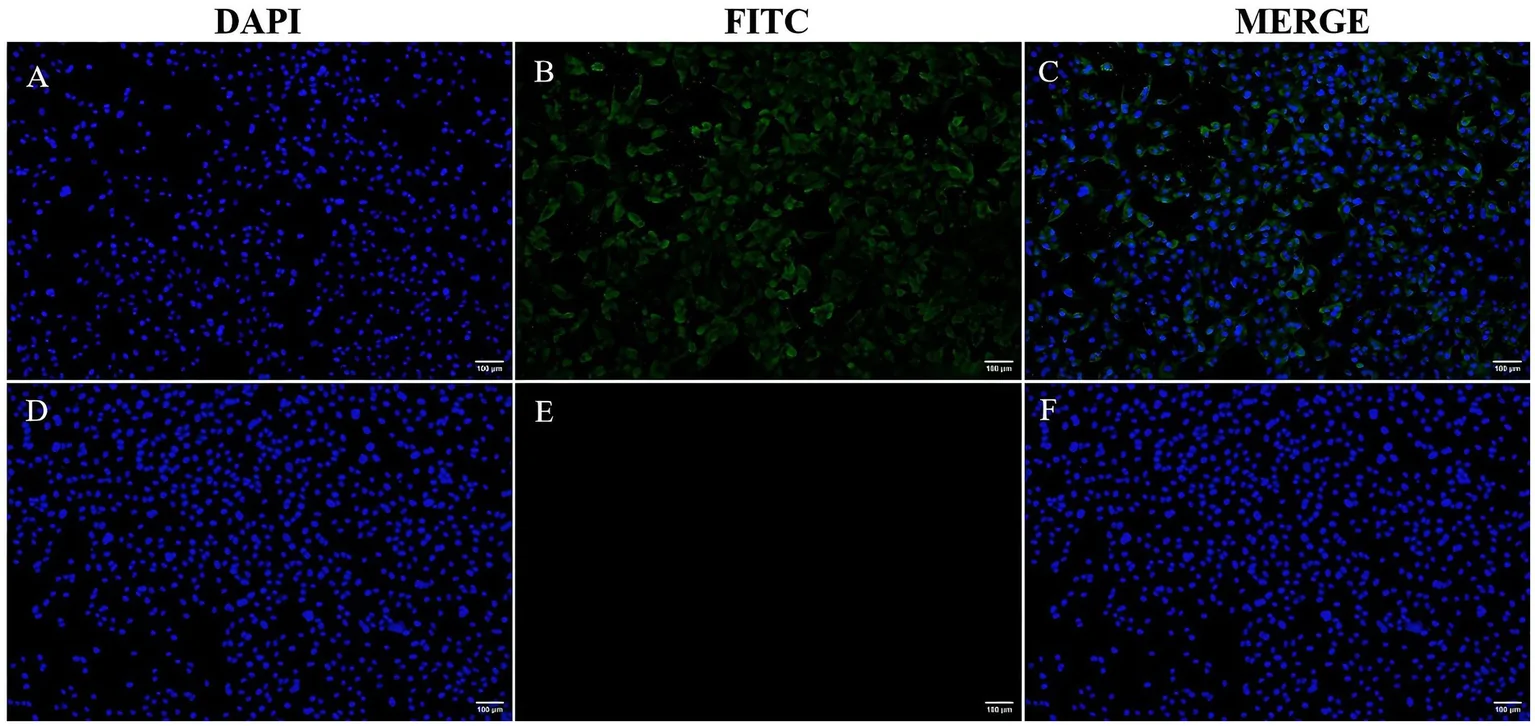
Detection of duck serum samples by IFA. **(A–C)** Vero cells infected with aMPV-FJ21 showed green fluorescence when incubated with anti-aMPV/C-positive duck sera. **(D–F)** No fluorescence signal was detected with negative sera.

**Table 2:** Detection of aMPV/C infection in four duck breeds *via* iELISA.

Duck breeds	Number of tested samples	Number of positive samples	Positivity rate (%)
Sheldrake ducks	136	94	69.1
Muscovy ducks	42	24	57.1
Cherry Valley ducks	38	11	28.9
Total	216	129	59.7

**Table 3:** Comparison of the self-established iELISA with IFA for detection of duck sera.

Serum samples	Self-established iELISA		
	Positive samples	Negative samples	Total
Positive samples tested by IFA	45	0	45
Negative samples tested by IFA	0	8	8
Total	45	8	53
Coincidence rate	100%		

## Discussion

Since Although aMPV subtype B has historically been the dominant lineage in Chinese poultry, the epidemiological landscape has shifted considerably since 2021, with aMPV/C now emerging as a significant pathogen in domestic duck populations (Fu et al., 2025; Xu et al., 2025; Yu et al., 2024; Zhang et al., 2022). This epidemiological transition is particularly noteworthy given that aMPV/C was first detected in ducks in 2014 but remained largely unreported in waterfowl for nearly a decade, suggesting that the virus may have undergone adaptive changes that facilitated its spread and establishment in duck hosts (Sun et al., 2014). The recent identification of a novel aMPV/C lineage in Sheldrake ducks is especially concerning, as this variant appears to have acquired a distinct tropism for reproductive tissues, manifesting as hydrosalpinx fluid syndrome rather than the classical respiratory disease typically associated with aMPV infections (Fu et al., 2025; Yu et al., 2024). This clinical shift from respiratory to reproductive pathology may reflect ongoing viral evolution and adaptation to new host species, potentially involving changes in the viral attachment protein or other surface glycoproteins that determine cell tropism. Furthermore, the seasonal recurrence of this disease on affected farms, often correlated with annual climatic variations, complicates disease control efforts and underscores the urgent need for diagnostic tools that are both reliable and broadly applicable across different duck breeds and production systems (Fu et al., 2025; Sun et al., 2014).

Historically, aMPV subtypes A, B, and C have predominantly circulated in chickens and turkeys worldwide (Cook, 2000; Salles et al., 2023; Wei et al., 2013). Consequently, numerous indirect ELISAs using various structural proteins (N, M, F, and G) as coating antigens have been developed for serological surveillance in these species (Chiang et al., 2000; Gulati et al., 2000; Gulati et al., 2001; Alvarez et al., 2004; Xu et al., 2021). However, no commercial ELISA kit is currently available for detecting aMPV antibodies in duck sera, particularly against subtype C. To address this diagnostic gap, we developed an iELISA using whole inactivated aMPV/C virions as the coating antigen. This approach offers distinct advantages over recombinant protein-based assays, which have been the standard for aMPV serology in chickens and turkeys. While recombinant proteins are widely adopted due to their ease of production and scalability, they often fail to present the full repertoire of conformational and linear epitopes present on native viral particles, and interference from components of the prokaryotic expression host (*Escherichia coli*), potentially compromising assay sensitivity and leading to false-negative results, especially in low-titer samples (Meireles et al., 2020; Xu et al., 2026). In contrast, whole-virion antigens preserve the native antigenic architecture, enabling more comprehensive detection across a broader range of antibody specificities and isotypes (Sumita et al., 2016). This feature is particularly valuable for detecting antibodies against emerging viral variants, as a single recombinant protein may not adequately capture the antigenic diversity of circulating strains. The aMPV-FJ21 strain used in this study achieved high titers in Vero cells, substantially exceeding previously reported levels at the same passage, thereby providing ample antigen for large-scale production while ensuring consistent quality (Sun et al., 2014; Yao et al., 2025; Wang et al., 2023). The use of a single master seed stock, combined with serum-free culture conditions and rigorous control of passage number and culture parameters, further ensured batch- to-batch consistency and minimized the risk of exogenous protein contamination that could otherwise compromise assay specificity.

The analytical performance of the iELISA was robust and met the requirements for a practical diagnostic tool. No cross-reactivity was observed against sera positive for nine other major duck pathogens, including H5 AIV, NDV, EDSV, DTMUV, DPV, BTV, MDPV, GPV, and DHAV-3, demonstrating high specificity. This specificity is critical for accurate diagnosis in field settings where co-infections with multiple pathogens are common. The detection limit of 1:102400 indicates high sensitivity, making the assay suitable for detecting low-titer antibodies in field samples, particularly during the early stages of infection or in vaccinated flocks where antibody levels may be relatively low. The excellent reproducibility of the assay, with coefficients of variation below 10% in both intra- and inter-assay evaluations, confirms its operational stability and reliability for routine diagnostic use across different laboratories and operators.

Application of the iELISA to 216 clinical serum samples collected from three duck breeds revealed an overall seroprevalence of 59.7%, with notable variation among breeds: Sheldrake ducks showed the highest positivity rate (69.1%), followed by Muscovy ducks (57.1%) and Cherry Valley ducks (28.9%). This breed-dependent variation may reflect differences in genetic susceptibility, management practices, or environmental exposure, and warrants further investigation into the host factors that influence aMPV/C infection dynamics. The higher seroprevalence observed in Sheldrake and Muscovy ducks compared to Cherry Valley ducks could be related to breed-specific differences in receptor expression, immune response efficiency, or husbandry practices that affect virus transmission. The complete concordance (100%) between the iELISA and IFA in a subset of 53 samples further validates the reliability of the assay and supports its utility as a practical tool for large-scale serological surveillance. This high level of agreement suggests that the iELISA can be used interchangeably with IFA, offering the advantages of higher throughput, objective readout, and reduced labor intensity, making it particularly suitable for epidemiological surveys and routine monitoring programs in commercial duck farms. These findings collectively support the suitability of the developed iELISA for large-scale serological surveillance of aMPV/C infection in duck flocks, providing a valuable tool for disease monitoring and control efforts.

## Conclusion

In summary, we developed a sensitive, specific, and reproducible iELISA for detecting aMPV/C antibodies in duck sera, demonstrating 100% concordance with IFA. This assay provides a practical tool for large-scale serological surveillance. The widespread seropositivity across duck breeds highlights the urgent need for vaccination strategies and routine monitoring to mitigate the impact of aMPV/C on China’s duck industry.

## Data Availability

The original contributions presented in the study are included in the article/supplementary material, further inquiries can be directed to the corresponding author/s.
